# Validation of a short version of the Lee fatigue scale in adults living in Norway: a cross-sectional population survey

**DOI:** 10.1186/s12889-023-17036-1

**Published:** 2023-10-31

**Authors:** Anners Lerdal, Caryl Gay, Tore Bonsaksen, Øivind Ekeberg, Trine Grimholt, Trond Heir, Anders Kottorp, Kathryn A. Lee, Laila Skogstad, Inger Schou-Bredal

**Affiliations:** 1 Research Department, Lovisenberg Diaconal Hospital, Oslo, Norway; 2 Department of Interdisciplinary Health Sciences, Faculty of Medicine, Institute of Health and Society, University of Oslo, Oslo, Norway; 3 Department of Family Health Care Nursing, University of California, San Francisco, USA; 4 Department of Health and Nursing, Faculty of Social and Health Sciences, Inland Norway University of Applied Sciences, Elverum, Norway; 5 Department of Health, Faculty of Health Studies, VID Specialized University, Stavanger, Norway; 6 Psychosomatic and CL Psychiatry, Division of Mental Health and Addiction, Oslo University Hospital, Oslo, Norway; 7 Department of Health, Faculty of Health Studies, VID Specialized University, Oslo, Norway; 8 Department of Acute Medicine, Oslo University Hospital, Oslo, Norway; 9 Norwegian Center for Violence and Traumatic Stress Studies, Oslo, Norway; 10 Institute of Clinical Medicine, University of Oslo, Oslo, Norway; 11 Faculty of Health and Society, Malmö University, Malmö, Sweden; 12 Faculty of Health Sciences, Department of Health and Care Sciences, UiT The Arctic University of Norway, Tromsø, Norway; 13 Department of Public Health Science, Faculty of Medicine, Institute of Health and Society, University of Oslo, Oslo, Norway

**Keywords:** Fatigue, Lee Fatigue Scale, Low energy, Psychometrics, Rasch analysis, Population survey

## Abstract

**Background:**

Due to the nature of fatigue, a brief reliable measure of fatigue severity is needed. Thus, the aim of our study was to evaluate a short version of the Lee Fatigue Scale (LFS) in the Norwegian general population.

**Methods:**

This cross-sectional survey consists of a representative sample from the Norwegian population drawn by The National Population Register in Norway. The study is part of a larger study (NORPOP) aimed at collecting normative data from several questionnaires focused on health in adults living in Norway. Registered citizens between 18 and 94 years of age were randomly selected stratified by age, sex and geographic region. Of the 4971 respondents eligible for the study, 1792 (36%) responded to the survey. In addition to age and sex, we collected responses on a 5-item version of the LFS measuring current fatige severity. The psychometric properties focusing on internal structure and precision of the LFS items were analyzed by a Rasch rating scale model.

**Results:**

Complete LFS scores for analyses were available for 1767 adults. Women had higher LFS-scores than men, and adults < 55 years old had higher scores than older respondents. Our analysis of the LFS showed that the average category on each item advanced monotonically. Two of the five items demonstrated misfit, while the three other items demonstrated goodness-of-fit to the model and uni-dimensionality. Items #1 and #4 (tired and fatigue respectively) showed differential item functioning (DIF) by sex, but no items showed DIFs in relation to age. The separation index of the LFS 3-item scale showed that the sample could be separated into three different groups according to the respondents’ fatigue levels. The LFS-3 raw scores correlated strongly with the Rasch measure from the three items. The core dimensions in these individual items were very similarly expressed in the Norwegian language version and this may be a threat to the cultural-related or language validity of a short version of the LFS using these particular items.

**Conclusions:**

The study provides validation of a short LFS 3-item version for estimating fatigue in the general population.

## Background

The Lee Fatigue Scale (LFS) [1] is frequently used to measure fatigue severity in a variety of patient populations. It has mostly been used to measure fatigue severity in patients with cancer [2–4], human immunodeficiency virus (HIV) [5], osteoarthritis [6, 7], stroke [7], obstructive sleep apnea [8], patients undergoing dialysis [9], and patients treated in intensive care units [10] as well as in pregnant women [11] and parents of preterm infants [12]. Several studies have shown that the LFS is sensitive in measuring diurnal patterns of fatigue, distinguishing between morning and evening levels of fatigue [3, 5, 13, 14].

When the psychometric properties of the original 13-item version of fatigue items on the LFS were evaluated with a Rasch analysis approach that compared fatigue scores obtained in the morning and evening [15], nine of the items showed satisfactory acceptable goodness-of-fit for the morning and 10 items for the evening measures. These items also demonstrated an acceptable level of uni-dimensionality and were able to differentiate the patients into four levels of fatigue severity.

Because of the nature of fatigue, it is important to reduce participant burden when conducting research on fatigue. Reducing the burden on participants is also relevant in the context of public health surveys, where participants are asked to respond to a broad range of questionnaires. A previous study found that most of the frequently used instruments to assess fatigue consisted of 10–20 items [16], which indicates that the development and validation of short fatigue instruments may be particularly useful. In a study that aimed to develop a short version of the LFS [17], a sub-set of five of the original 13 items was found to be sufficient to measure fatigue severity and satisfy criteria for internal scale validity, uni-dimensionality and separation of patients’ fatigue into three distinct levels, which is sufficient for many clinical and research purposes. A short version in Norwegian of the LFS has been used in clinical studies of Norwegian patients, but the psychometric properties has not been tested in the Norwegian general population. This is need in order to know if normative data from the general population can be used as reference values in relation to LSF scores in clinical studies. Furthermore, the LFS, in contrast to other fatigue scales, has been used for two daily measurements over several days to describe morning and evening fatigue [3, 5, 18]. Since the patients have to fill in the LFS so frequently, it is of particular interest to have a valid and reliable short version of the instrument.

Although fatigue is often studied in chronic illness populations, several studies show that fatigue is also experienced in the general population, including people without current diseases. Depending on the definition and cut-off value for fatigue cases, the prevalence of current fatigue in the general population has been reported to vary between 5 and 30% [19–21], and the prevalence of chronic fatigue (lasting more than six months) has ranged from 6 to 30% [19, 20, 22]. Given the large variation in prevalence between these studies the validity of cut-off scores designating a positive case of fatigue is of concern. Due to the nature of fatigue, a short fatigue instrument that requires little energy for respondents to complete and with satisfactory psychometric properties is warranted to survey fatigue in the general population. It is important that fatigue measures be validated for use not only in various patient populations, but in the general population as well. No previous validation studies of the LFS in the general population exist. Thus, the aim of this study was to examine the psychometric properties focusing on internal structure and precision [23] of a short version of the LFS in the general population in Norway.

## Methods

A representative sample of the Norwegian population was surveyed in a cross-sectional study in order to establish normative data for a number of different instruments measuring different symptoms, health behaviors and attitudes. The National Population Register in Norway drew a representative sample of the Norwegian population. All registered citizens in Norway between 18 and 94 years of age were eligible to participate, and the sample was stratified according to age, sex, and geographic region. A total of 5500 citizens were invited to participate, including citizens from all of the country’s 19 counties. A more detailed description of the recruitment process has been published elsewhere [24, 25].

### Procedures

The sample was mailed the questionnaires in 2015 with information about the study and a pre-paid return envelope. Each individual was mailed two reminders. Of the 4,971 survey recipients, 1,792 (36%) returned their survey. Between responders and non-responders, there were no significant differences in mean age, gender proportions or proportions living in rural versus urban areas [25]. The proportion of the sample in active work was 66%, compared to 67% in the general population [26]. Among responders and non-responders alike, 17% lived alone. Among responders, 1.3% were without work and 53% had higher education, compared to 4.4% and 41.0% in the general population, respectively [24]. In view of these comparisons, the sample was deemed fairly representative of the general Norwegian population.

### Measurements

Fatigue severity was measured with a 5-item version of the LFS [1]. Each of the items has two anchor statements, and participants responded on an 11-point numeric rating scale, with responses ranging from 0 to 10. The five items anchor statements were: *not at all tired – extremely tired* (item #1), *not at all fatigued – extremely fatigued* (item #4), *not at all worn out – extremely worn out* (item #5), *not at all bushed – extremely bushed* (item #11), and *not at all exhausted – extremely exhausted* (item #12). The item numbers refer to the original LFS version [1]. The mean score of the 5 item scores constitutes each participant’s fatigue severity score. Demographic information on age (in years) and sex (male, female) was also collected.

### Ethics

The Regional Committee for Medical and Health Research Ethics South East was consulted prior to distributing the survey. Because this was an anonymous survey conducted by mail, the committee did not require formal review of the study. Returned surveys signified consent to participate.

### Statistical analysis

Descriptive statistics were used to summarize sample demographics and fatigue scores.

The psychometric analysis of the LFS was guided by a Rasch rating scale model [27]. The transformed 11-category raw scores (0 to 10) from the five LFS items were analyzed using the WINSTEPS Rasch computer software program, version 3.91.0.0 [28]. The analyses were performed using a systematic stepwise approach similar to that used in previous studies [5].

In *Step 1*, an evaluation of the psychometric properties of the fatigue rating scale was conducted to determine whether the average measures for each category on each item advanced monotonically, i.e. whether the Outfit Mean Square *(MnSq)* values were < 2.0 for each of the step calibrations [29].

*Step 2* aimed to evaluate the fit of the item responses [27]. Any item that did not show acceptable goodness-of-fit to the model was removed, and the psychometric properties of the remaining items were re-analyzed until all remaining items demonstrated acceptable goodness-of-fit. For this study, acceptable goodness-of-fit was defined as *Infit MnSq* values between 0.7 and 1.3, which is stricter than the suggested guidelines for surveys using rating scales [30]. We choose to focus on infit statistics in this study as they are considered the most informative measure of goodness-of-fit given that they focus on the degree of fit in the most typical observations in the data [31]. We also focus on *MnSq* values rather than standardized *z*-values, as z-values are highly influenced by sample size [32]. In *Step 2*, we also evaluated the level of uni-dimensionality in the generated LFS measure by a principal component analysis (PCA) of the residuals, with the criterion that the first latent dimension should explain at least 50% of total variance [33]. We also monitored the standardized item residual correlations between items using the Winsteps output and considered a correlation coefficient between items of 0.5 or higher (equal to a shared variance of 25% or more) to be a threat to local independence.

*Step 3* evaluated aspects of person response validity. Person goodness-of-fit was defined as *Infit MnSq* values less than 1.4 logits or associated with a z-value < 2, accepting that 5% of the sample may by chance fail to demonstrate acceptable goodness-of-fit without threatening evidence of person response validity [34–36].

In *Step 4*, differential item functioning (DIF) analyses were performed in order to evaluate the stability of the LFS response patterns in relation to age and sex using the Mantel–Haenszel statistics for polytomous scales using log-odds estimators [37, 38] as reported from the WINSTEPS program (*p* < 0.01 with Bonferroni correction). If DIF was detected for an item, a supplementary analysis of the impact of the item’s DIF was then calculated, based on a standardized *z*-comparison of the individual Rasch measures produced by the generic vs the sample-specific item hierarchies. Item DIF was considered to have minimal impact if 5% or less of the sample had a change of more than ± 1.96 z-value in their Rasch measures.

*Step 5* assessed several aspects of the fatigue scale’s reliability. The unidimensional scale’s ability to separate participants into distinct groups was estimated using the person separation index. The Rasch-equivalent person reliability coefficient, as well as the Cronbach’s alpha reliability coefficient based on the LFS raw item scores, were also reported for the final unidimensional scale. Finally, a Pearson’s correlation coefficient was used to evaluate the relationship between the LFS mean raw score (calculated as the mean of the item raw scores) and the Rasch-generated measures.

## Results

### Fatigue in the general population

Of the 1792 survey respondents, 1767 had complete LFS scores for analysis. The mean age of respondents was 53.2 years (± 16.6 SD), with a range 18–94 years. Fewer men (46.9%) responded compared to women (53.1%). As shown in Table 1, women had a higher mean LFS score than men (*p* < 0.001), and adults < 55 years old had a higher mean LFS score than adults 55 years and older (*p* < 0.001). Descriptive statistics for each of the LFS items are shown in Table 2.

**Table 1:** Lee Fatigue Scale (LFS) mean scores for men and women (*n* = 1767)

Characteristic	% (n)	LFS score mean (SD)	*p*-value
Total sample	100% (1767)^a^	3.33 (2.54)	
Sex	99% (1755)^b^		< .001
Men	47% (822)	2.99 (2.38)	
Women	53% (933)	3.65 (2.64)	
Age	99% (1748)^c^		< .001
18–54 years	50% (874)	3.60 (2.60)	
≥ 55 years	50% (874)	3.07 (2.46)	

^a^25 of the 1792 respondents answered < 4 LFS items, so a mean score was not computed

^b^13 of the 1767 respondents with LFS scores had missing data for self-identified gender

^c^19 of the 1767 respondents with LFS scores had missing data for age

**Table 2:** Descriptive statistics for five LFS items (*n* = 1767^a^)

Item	Missing(n)	Mean (SD)	% with minimum score of 0	% with maximum score of 10
1. Tired	0	4.51 (2.69)	8.1%	3.1%
4. Fatigued	0	3.25 (2.84)	22.9%	2.2%
5. Worn out	4	3.30 (2.82)	21.7%	2.0%
11. Bushed	1	2.95 (2.73)	26.4%	1.8%
12. Exhausted	3	2.66 (2.74)	31.7%	1.8%

^a^25 participants were excluded from the analysis because they were missing data on > 1 item

### Rasch analysis of LFS psychometric properties

Table 3 summarizes the findings of the Rasch analysis. In Step 1, the rating scale of the LFS demonstrated acceptable outcomes in relation to the set criteria. When analyzing the infit mean square statistics for the five items in *Step 2*, two items (items #1 tired and #5 worn out) demonstrated unacceptable fit statistics (see Table 3). We excluded these items and re-ran the analysis with the remaining three items. In the second iteration, all three remaining items demonstrated acceptable goodness-of-fit to the model. The uni-dimensionality of the 3-item LFS scale was also acceptable (83.6%).

**Table 3:** Overview of the Statistical Approach, Criteria, and Results of the Rasch Analysis of the LFS short form used in the general population (*n* = 1767)

Aspect of validity evidence measured	Statistical approach and criteria	Initial LFS short form scale (5 items)	Final LFS short form scale (3 items)	Comparison LFS short form scale (Bragstad et al. 2020) [7] (3 items)
***Step 1*:Internal scale validity**	**Rating scale functioning**	All criteria met	All criteria met	All criteria met
	The average measures for each response category should advance monotonically associated with outfit *MnSq* values < 2.0 for each of the step calibrations			
***Step 2*:Internal scale validity**	**Item goodness-of-fit statistics**	#12 exhausted: *MnSq* = *1.29*	#12 exhausted: *MnSq* 1.04	#5worn out: *MnSq* 0.87
	A sample-size adjusted criterion for item goodness-of-fit requiring infit *MnSq* values between 0.7 and 1.3			
				#1 tired: *MnSq* 1.16
		#11 bushed: *MnSq* = *0.99*	#11 bushed: *MnSq* 0.86	#4 fatigued: *MnSq* 0.87
			#4 fatigued: *MnSq* 1.05	
		#5 worn out: *MnSq* = 0.64		
		#1 tired: *MnSq* = *1.33*		
		#4 fatigued: *MnSq* = *0.77*		
	**Principal component analysis of the residuals**	83.3%	83.6%	88.1%
	The criterion was set for at least 50% of the total variance to be explained by the first latent variable	The largest standardized residual correlation coefficient was 0.02 between the items #4 Bushed and #5 Exhausted	All standardized item residual correlation coefficients were negative	Not reported
	**Local independence among items**			
	The criterion was set as all correlation coefficients between item residuals should be 0.5 or less (equal to a shared variance of 25% or less)			
***Step 3*: Person response validity**	**Person goodness-of-fit statistics**	-	113 (6.4%)	86 (4.9%)
	A criterion of < 5% of the sample has unacceptable person goodness-of-fit, as indicated by infit *MnSq* values lower than 1.4 associated with a standardized z value < 2.0			
***Step 4*: Item stability**	**Differential item functioning (DIF)**	-	Gender: None	Gender:
	No item DIF should exceed a criterion of* p* = .01			
			Age: None	Item #4 Fatigued: less challenging to endorse for men (mean of 54.62 logits) than for women (mean of 56.38 logits, p < .01)
				Item #1 Tired: Less challenging to endorse for women (mean of 38.57 logits) than for men (mean of 40.83 logits, p < .01)
				Age: None
***Step 5*: Test reliability**	**Person-separation reliability**			
	Person-separation index > 2.0 was required to ensure that the scale could differentiate the sample in at least three different levels of fatigue interference	Separation index = 3.45	Separation index = 2.59	Separation index = 3.55
		Correlation between raw score and Rasch measure = .96	Correlation between raw score and Rasch measure = .97	Correlation between raw score and Rasch measure = .97
		Person reliability (Rasch) = .92	Person reliability (Rasch) = .87	Person reliability (Rasch) = .93
		Cr-α (raw score) = .95	Cr-α (raw score) = .93	Cr-α (raw score) = .94
	**Person reliability (Rasch) Cronbach’s alpha coefficient for raw scores**			
		Maximum score = 20 (1.1%)	Maximum score = 20 (1.1%)	Maximum score = 29 (1.6%)
		Minimum score = 129 (7.3%)	Minimum score = 319 (18.0%)	Minimum score = 135 (7.6%)

*MnSq* Mean square

In *Step 3*, a proportion of the sample close to the set criteria demonstrated misfit to the Rasch model in the 3-item LFS scale (6.4%). In *Step 4*, none of the three items demonstrated DIF in relation to Gender or Age (using median splits), so no supplementary analysis of the impact of item DIF was performed.

In *Step 5*, the separation index of the LFS scale decreased (from 3.45 to 2.49) after deleting the two items demonstrating misfit in Step 2, but still exceeded our set criterion. There was a strong correlation between the LFS raw score and the Rasch measure, which also remained stable (from 0.96 to 0.97) after deleting the two misfitting items. Person reliability for the Rasch measure and Cronbach’s alpha for the raw score were also acceptable but decreased after deleting the two misfitting items. Given the proportions of minimum and maximum scores, there was a minimal ceiling effect, but some evidence of a floor effect, which worsened after deleting the two misfitting items (from 7.3% to 18.0% having minimum scores).

## Discussion

In this population-based study, a 3-item version of the Lee Fatigue Scale demonstrated acceptable psychometric properties for assessing fatigue in the general population. These findings provide further evidence for the psychometric properties of the LFS and support prior studies where we have shown that short versions of the LFS have satisfactory internal scale validity, uni-dimensionality and sensitivity to separate individuals with fatigue into three different groups (low, medium and high levels of fatigue) [17, 39]. Thus, a shorter measure of fatigue severity may be as psychometrically sound as longer measures. This is particularly important for measures of fatigue where the level of fatigue may impact on the respondents’ ability and capacity to participate in fatigue studies. While previous studies have assessed the psychometric properties of the LFS-VAS with scores converted from the 100 mm VAS to numeric scores from 0–10, the present study showed that the average measures for each response category advanced monotonically. The floor effect of the LFS-3 item was relatively high and higher than in previous studies using other versions of the LFS. However, this can be explained by the fact that the current sample represents the general population where fatigue is expected to be considerably lower compared to samples of clinical patients with acute or chronic illness. Thus, the floor effect in this study may not be considered a psychometric weakness, as it is expected that not everyone in the population would experience even a low perceived level of fatigue. While a previous psychometric assessment of the LFS was performed in a sample of women, this current study included men and demonstrated that two of the items from LFS measure showed DIFs biased by sex, i.e. women endorsed “fatigue” more easily than men, while men endorsed “tired” more easily than women.

The core dimension of fatigue severity used in the 5-item English language LFS is reflected in the similar but unique wording of items (tired, fatigued, worn out, bushed and exhausted). The same core dimension may not be reflected when using Norwegian words with very similar meanings. Language may indeed impact the cultural validity of a short Norwegian version of LFS with the particular combination of items used in our study. Conceptual translations for cross-cultural comparisons are complex and would require additional studies to ensure that items or concepts in different languages address the same construct.

Slightly higher levels of fatigue than expected were found in persons demonstrating misfit to the Rasch model. More data may be needed here to assess whether any systematic pattern among the persons demonstrating misfit can be detected and allow for specific subgroup analysis. Earlier studies using the LFS have shown item calibration differences between diagnostic groups [7], which may be hidden in general adult population samples, such as the sample used in this study.

Some of the items had a relatively high proportion of minimum scores. However, since our study surveyed fatigue in the general population, we expected that a large proportion did not experience fatigue.

A significant challenge for building cumulative knowledge of fatigue in different groups of patients and populations is the absence of consensus among researchers about which fatigue instrument should be used. There are several promising initiatives to develop international recommendations for measuring fatigue and psychometrically sound instruments that are available in many languages, such as the 13-item PROMIS SF v1.0 Fatigue Scale [40]. While such measures may have some advantages, the psychometrically valid 3-item LFS may be more suitable when a shorter scale is needed.

### Strengths

A relatively large sample drawn from the general population provides evidence of validity of a short three-item version of the LFS that is also sensitive enough to differentiate the sample into three distinct groups by level of fatigue.

### Limitations

Although our findings in a Norwegian version of the LFS are similar to those in the English version, caution in interpreting the findings is warranted. Translating fatigue and similar symptoms from the original English version is difficult and can be problematic when attempting to generalize our findings to other non-English speaking cultures. Even American English could have subtle differences in meaning compared to British English wording for items in the LFS. The Norwegian LFS items evaluated in this study originate from a translation done for a prior study with a diagnosis-specific female sample [41]. In addition, the translation process was not performed according to the current standards described in the COSMIN framework [42]. Furthermore, Since the survey did not include the full original Lee Fatigue Scale we only had the opportunity to validate this short version based on data generated using the pre-selected items. Thus, the generalization and applicability of the LFS scale in general to a wider population can be questioned. With a limited number of fatigue items in this study, there is also a risk that some other LFS items, now not included, could have demonstrated acceptable goodness-of-fit to the Rasch model. This could also impact on evidence based on test content, potentially missing additional essential aspects of fatigue. Various validity studies of the LFS scale have also suggested different item combinations to measure the level of fatigue in a valid manner [17, 39]. This can be viewed as a limitation especially when comparing outcomes from different studies using different item combinations. A solution to this challenge could be to apply a Rasch analysis model to generate a stable item bank or item hierarchy across large samples with diversity in diagnoses and languages that demonstrate acceptable validity, evidence of stability and internal construct validity. Providing the generic weights of each item calibration can then be used to select and use subsets of items for specific studies and samples, and still generate comparable measures. Such systematic approach should also be better grounded in the COSMIN guidelines regarding the full validation process, including translation.

## Conclusions

We surveyed Norwegian adults across the age spectrum to evaluate the psychometric properties of a Norwegian version of the LFS. Our results provide evidence for the validity of a short three-item version of the tool. Therefore, this version of the instrument can be readily applied to measure levels of fatigue in the general Norwegian population.

## Data Availability

The datasets used and/or analyzed during the current study are available from the corresponding author on reasonable request.

## Abbreviations

DIF: Differential Item Functioning
HIV: Human Immunodeficiency Virus
LFS: Lee Fatigue Scale
NORPOP: The Norwegian Population Study
MnSq: Mean Square
VAS: Visual Analogue Scale

## References

[1] Lee KA, Hicks G, Nino-Murcia G (1991). Validity and reliability of a scale to assess fatigue. Psychiatry Res.

[2] Golan-Vered Y, Pud D (2013). Chemotherapy-induced neuropathic pain and its relation to cluster symptoms in breast cancer patients treated with paclitaxel. Pain Pract.

[3] Lin Y, Bailey DE, Xiao C, Hammer M, Paul SM, Cooper BA, Conley YP, Levine JD, Kober KM, Miaskowski C (2022). Distinct Co-occurring Morning and Evening Fatigue Profiles in Patients With Gastrointestinal Cancers Receiving Chemotherapy. Cancer Nurs.

[4] Hugoy T, Lerdal A, Rustoen T, Oksholm T (2019). Predicting postoperative fatigue in surgically treated lung cancer patients in Norway: a longitudinal 5-month follow-up study. BMJ Open.

[5] Lerdal A, Gay CL, Aouizerat BE, Portillo CJ, Lee KA (2011). Patterns of morning and evening fatigue among adults with HIV/AIDS. J Clin Nurs.

[6] Lindberg MF, Miaskowski C, Rustoen T, Rosseland LA, Paul SM, Cooper BA, Lerdal A (2017). The Impact of Demographic, Clinical, Symptom and Psychological Characteristics on the Trajectories of Acute Postoperative Pain After Total Knee Arthroplasty. Pain Med.

[7] Bragstad LK, Lerdal A, Gay CL, Kirkevold M, Lee KA, Lindberg MF, Skogestad IJ, Hjelle EG, Sveen U, Kottorp A (2020). Psychometric properties of a short version of Lee Fatigue Scale used as a generic PROM in persons with stroke or osteoarthritis: assessment using a Rasch analysis approach. Health Qual Life Outcomes.

[8] Yang H, Engeland CG, King TS, Sawyer AM (2020). The relationship between diurnal variation of cytokines and symptom expression in mild obstructive sleep apnea. J Clin Sleep Med.

[9] Chao CT, Huang JW, Chiang CK (2016). group Cs: Functional assessment of chronic illness therapy-the fatigue scale exhibits stronger associations with clinical parameters in chronic dialysis patients compared to other fatigue-assessing instruments. PeerJ.

[10] Day A, Haj-Bakri S, Lubchansky S, Mehta S (2013). Sleep, anxiety and fatigue in family members of patients admitted to the intensive care unit: a questionnaire study. Crit Care.

[11] Tsai SY, Shun SC, Lai YH, Lee YL, Lee SY (2014). Psychometric evaluation of a Chinese version of the Lee Fatigue Scale-Short Form in women during pregnancy and postpartum. Int J Nurs Stud.

[12] Nordheim T, Rustoen T, Iversen PO, Nakstad B (2016). Quality of life in parents of preterm infants in a randomized nutritional intervention trial. Food Nutr Res.

[13] Aouizerat BE, Dhruva A, Paul SM, Cooper BA, Kober KM, Miaskowski C: Phenotypic and Molecular Evidence Suggests That Decrements in Morning and Evening Energy Are Distinct but Related Symptoms. J Pain Symptom Manage 2015, 50(5):599–614 e593. 10.1016/j.jpainsymman.2015.05.008 10.1016/j.jpainsymman.2015.05.008 PMC4624028 26031709

[14] Wright F, D'Eramo Melkus G, Hammer M, Schmidt BL, Knobf MT, Paul SM, Cartwright F, Mastick J, Cooper BA, Chen LM (2015). Predictors and Trajectories of Morning Fatigue Are Distinct From Evening Fatigue. J Pain Symptom Manage.

[15] Lerdal A, Kottorp A, Gay C, Aouizerat BE, Lee KA, Miaskowski C (2016). A Rasch Analysis of Assessments of Morning and Evening Fatigue in Oncology Patients Using the Lee Fatigue Scale. J Pain Symptom Manage.

[16] Hjollund NH, Andersen JH, Bech P (2007). Assessment of fatigue in chronic disease: a bibliographic study of fatigue measurement scales. Health Qual Life Outcomes.

[17] Lerdal A, Kottorp A, Gay CL, Lee KA (2013). Development of a short version of the Lee Visual Analogue Fatigue Scale in a sample of women with HIV/AIDS: a Rasch analysis application. Qual Life Res.

[18] Kober KM, Harris C, Conley YP, Dhruva A, Dokiparthi V, Hammer MJ, Levine JD, Oppegaard K, Paul S, Shin J (2022). Perturbations in common and distinct inflammatory pathways associated with morning and evening fatigue in outpatients receiving chemotherapy. Cancer Med.

[19] van't Leven M, Zielhuis GA, van der Meer JW, Verbeek AL, Bleijenberg G: Fatigue and chronic fatigue syndrome-like complaints in the general population. Eur J Public Health 2010, 20(3):251–257. 10.1093/eurpub/ckp113 10.1093/eurpub/ckp113 19689970

[20] Loge JH, Ekeberg O, Kaasa S: Fatigue in the general Norwegian population: normative data and associations. J Psychosom Res 1998, 45(1 Spec No):53–65. 10.1016/s0022-3999(97)00291-2 10.1016/s0022-3999(97)00291-2 9720855

[21] Lerdal A, Wahl A, Rustøen T, Hanestad BR, Moum T (2005). Fatigue in the general population: a translation and test of the psychometric properties of the Norwegian version of the Fatigue Severity Scale. Scand J Public Health.

[22] Martin A, Chalder T, Rief W, Braehler E (2007). The relationship between chronic fatigue and somatization syndrome: a general population survey. J Psychosom Res.

[23] American Educational Research Association (2014). American Psychological Association, National Council on Measurement in Education: Standards for Educational and Psychological Testing.

[24] Schou-Bredal I, Heir T, Skogstad L, Bonsaksen T, Lerdal A, Grimholt T, Ekeberg O (2017). Population-based norms of the Life Orientation Test-Revised (LOT-R). Int J Clin Health Psychol.

[25] Bonsaksen T, Lerdal A, Heir T, Ekeberg O, Skogstad L, Grimholt TK, Schou-Bredal I (2019). General self-efficacy in the Norwegian population: Differences and similarities between sociodemographic groups. Scand J Public Health.

[26] Statistics Norway: Labour force survey, Q4. In.; 2016. https://www.ssb.no/en/arbeid-og-lonn/statistikker/aku.

[27] Bond TG, Fox CM (2007). Applying the Rasch model: fundamental measurement in the human sciences.

[28] Linacre JM: Winsteps - Rasch Model computer program (Version 3.9.1.0). In. Chicago: www.winsteps.com; 2016.

[29] Linacre JM (2002). Optimizing rating scale category effectiveness. J Appl Meas.

[30] Wright BD, Linacre JM (1994). Reasonable mean-square fit values. Rasch Measurement Transactions.

[31] McNamara TF (1996). Measuring second language performance.

[32] Smith AB, Rush R, Fallowfield LJ, Velikova G, Sharpe M (2008). Rasch fit statistics and sample size considerations for polytomous data. BMC Med Res Methodol.

[33] Linacre JM (2011). A User׳s Guide to Winsteps. Ministep Rasch-Model Computer Programs. Program Manual 3.73.0.

[34] Kottorp A, Bernspang B, Fisher AG (2003). Validity of a performance assessment of activities of daily living for people with developmental disabilities. J Intellect Disabil Res.

[35] Patomella AH, Tham K, Kottorp A (2006). P-drive: assessment of driving performance after stroke. J Rehabil Med.

[36] Hallgren M, Nygard L, Kottorp A (2011). Technology and everyday functioning in people with intellectual disabilities: a Rasch analysis of the Everyday Technology Use Questionnaire (ETUQ). J Intellect Disabil Res.

[37] Mantel N (1963). Chi-square tests with one degree of freedom; Extensions of the Mantel-Haenszel procedure. J Am Stat Assoc.

[38] Mantel N, Haenszel W (1959). Statistical aspects of the analysis of data from retrospective studies of disease. J Natl Cancer Inst.

[39] Lerdal A, Kottorp A, Gay CL, Lee KA (2013). Lee Fatigue And Energy Scales: exploring aspects of validity in a sample of women with HIV using an application of a Rasch model. Psychiatry Res.

[40] PROMIS SF v1.0 Fatigue 13a [https://www.facit.org/measures/PROMIS-SF-v1.0-Fatigue-13a]

[41] Schjolberg TK, Dodd M, Henriksen N, Asplund K, Cvancarova Smastuen M, Rustoen T (2014). Effects of an educational intervention for managing fatigue in women with early stage breast cancer. European journal of oncology nursing : the official journal of European Oncology Nursing Society.

[42] COSMIN Study Design checklist for Patient-reported outcome measurement instruments [https://www.cosmin.nl/wp-content/uploads/COSMIN-study-designing-checklist_final.pdf]

